# Women and Emigration

**Published:** 1920-01-24

**Authors:** 


### WOMEN AND EMIGRATION.

### By a SOCIAL WORKER.

The ex-V.A.D., the nurse whose job has ended with the closing of hospital or convalescent home, the demobilised W.R.N.S., W.A.A.C., and W.R.A.F. are in many cases resting tired limbs gladly, but finding it very difficult to rest in mind. The thought of the future will obtrude itself anxiously. Some cannot afford to be without paid occupation; some—and these are many—feel that they cannot go home and hang their hands in the old idleness and emptiness. They have, perhaps, a small home, which does not of itself furnish occupations and duties, especially if there are several daughters and the mother is perfectly able-bodied and mentally competent. She does not need to be tucked up on sofas and waited upon. Very probably she plays golf; and while the girl was doing war work she became entirely accustomed to making her own life. Some playtime together? Yes, by all means. But she, too, is anxious not to see her daughter dawdle on in the uncertain hope that some man will come to wind up and set going for her the clock of daily duty.

So, to change the metaphor, a great game of musical chairs is in progress. So many seats are free, those who drop into them leave the others to stand. The problem is, however, in this form of the game, how to bring chairs enough for all.

The re-settlement of women must be treated as a question for the Empire. The balance of the sexes needs to be redressed in many parts of that Empire, and the square pegs must not be fitted into round holes. Otherwise new problems of sickness, poverty, and overcrowding, with immorality and all its consequences, will merely be created.

A fine step in furthering emigration for suitable women and to suitable places has just been taken by the amalgamation of a number of societies undertaking this work into a single body called the Society for the Overseas Settlement of British Women. This Society held recently a first meeting at Bedford College, when a very large number of people, especially of young women to whom information might be of vital importance, attended.

Incidentally, let us digress to another question. In these days, when practical reconstructive work needs to be carried out without haste, but also without delay, can we not decide to reconstruct our meetings? Can we not eliminate from speeches all those wasted words in which speakers tell us that they are not really speakers, that they have really nothing to say, that they are taking the place of someone else whom we should all have preferred to hear, and that they are not going to keep us for many minutes? This particular meeting occupied nearly two and a-quarter hours; it had the benefit of addresses from Miss Gladys Pott, who has recently been to Canada as Commissioner to make inquiries; from Mrs. Hughes, whose husband's work in Australia has made him famous; and of two ladies speaking from long personal knowledge of conditions in New Zealand and South Africa. That the desire for concrete knowledge was very strong was shown by the large number of written questions sent up at the end of the meeting, no opportunity having been given at the end of each paper. When summarised, the actual practical information was, after all, rather scanty. But a few outstanding facts will help women who are pondering the question of personal emigration.

First, a consultation by letter or personal visit to the Society's office, Brompton Road, S.W., will be a very wise step to take. Secondly, it becomes more and more clear that there are favoured spots where women with certain qualifications have excellent prospects. Thus in South Africa the skilled violinist, the capable dressmaker and milliner, and in certain towns the social worker are urgently needed, and there are openings for qualified medical women, for teachers, for flower farmers, and for some research work in public health and agriculture departments. On the other hand, there is no chance for the masseuse or the tea-room manageress. In New Zealand the "lady help" is likely to be overtasked and perhaps underpaid, while the genuine domestic servant can earn £80 or £100, and have "a good time" with a trade union to back her up, and her passage if she undertakes to keep her place for two years. Specialised and well-qualified teachers can work up good connections. Australia, again, offers great opportunities for domestic service, but marriage must be counted on as possible rather than certain.

From this and every similar meeting one fact emerges, and women must reckon with it whether they wish to or not. It is that everywhere in the world the much-needed woman is the one who, whether by organisation, instruction, and superintendence of others, or by her own labour, can and will turn mere houses into homes. There are women who create home wherever they may happen to be; there are others who would turn the most perfectly constructed human nest into something suggestive of a hotel or a railway-station waiting-room. Like every other instinct, this one can be cherished and developed or repressed and withered. The best housing scheme in the world will prove futile unless parallel with their development runs the effort to direct the education of girls of all classes towards developing this side of their character. It is not a simple problem with a single answer, but one which must be worked at in every department of life, and not least—paradoxical as it may seem—in specialised and professional training. Is it not safe to say that women doctors are rather noted for the comfort and happiness of their private homes?

